# Endodormancy Release Can Be Modulated by the GA_4_-GID1c-DELLA2 Module in Peach Leaf Buds

**DOI:** 10.3389/fpls.2021.713514

**Published:** 2021-09-27

**Authors:** Sen Li, Qingjie Wang, Binbin Wen, Rui Zhang, Xiuli Jing, Wei Xiao, Xiude Chen, Qiuping Tan, Ling Li

**Affiliations:** ^1^College of Horticulture Science and Engineering, Shandong Agricultural University, Tai'an, China; ^2^State Key Laboratory of Crop Biology, Shandong Agricultural University, Tai'an, China; ^3^Shandong Collaborative Innovation Center for Fruit & Vegetable Production With High Quality and Efficiency, Tai'an, China; ^4^College of Life Sciences, Shandong Agricultural University, Tai'an, China

**Keywords:** *Prunus persica*, leaf bud endodormancy release, GA INSENSITIVE DWARF1 GID1c, DELLA protein, GA4

## Abstract

Gibberellin (GA) plays a key role in the release of bud dormancy and the GA receptor GID1 (GIBBERELLIN INSENSITIVE DWARF1) and DELLA protein are the GA signaling parts, but the molecular mechanism of GA-GID1-DELLA module regulating leaf bud dormancy in peach (*Prunus persica*) is still not very clear. In this study, we isolated and characterized the GID1 gene *PpGID1c* from the peach cultivar “Zhong you No.4.” Overexpressing *PpGID1c* in *Arabidopsis* promoted seed germination, which indicated that *PpGID1c* has an important function in dormancy. The expression level of PpGID1c in peach leaf buds during endodormancy release was higher than that during ecodormancy and was positively correlated with GA_4_ levels. Our study also found that GA_4_ had the most obvious effect on promoting the bud break, indicating that GA_4_ may be the key gibberellin to promoting peach leaf bud endodormancy release. Moreover, a quantitative real-time PCR (qRT-PCR) found that GA_4_ could increase the expression of the gibberellin signaling gene *PpDELLA2*. A yeast two-hybrid (Y2H) assay suggested that the PpGID1c interaction with the PpDELLA1 protein was not dependent on gibberellin, while the PpGID1c interaction with PpDELLA2 required GA_4_ or another gibberellin. These findings suggested that the GA_4_-GID1c-DELLA2 module regulates peach leaf bud endodormancy release, with this finding significantly enhancing our comprehensive understanding of bud endodormancy release and revealing a new mechanism for regulating leaf bud endodormancy release in peach.

## Introduction

In temperate and boreal regions, perennial plants have seasonal cycles of growth and dormancy to survive during the winter cold (Tylewicz et al., 2018). Accordingly, a study by Lang (1987) divided the stages of bud dormancy into paradormancy, endodormancy, and ecodormancy. This classification has been widely accepted by researchers in the study of bud dormancy. Paradormancy is the dormancy caused by the structure of the plant itself. It is affected by neighboring organs or tissues, which inhibit the growth of the plant. Endodormancy, also known as internal dormancy, is a dormant phenomenon controlled by its own internal factors. In this stage, only when dormant buds meet a certain low-temperature accumulation can they burst under appropriate conditions. Ecodormancy is a phenomenon in which plants cannot grow due to external environmental factors, such as growth stagnation caused by natural environmental stresses like low temperature and drought (Horvath et al., 2003). The bud dormancy associated with winter is generally called endodormancy (Wang et al., 2015). Bud endodormancy is essential for woody plants to resist the cold environment of winter. Recently, deciduous fruit trees have been incompletely flowering due to global warming and higher temperatures, thereby reducing yields and ultimately affecting the economic returns of fruit farmers. Therefore, understanding the regulatory mechanism of bud endodormancy is important for a better grasp of the flowering and fruiting of agricultural production and the improving of the yield and quality of fruits (Tuan et al., 2017).

Gibberellins are believed to play a key role in regulating bud dormancy. Gibberellin content determines the timing of the endodormancy release of pear buds (Yang et al., 2019). During Japanese apricot bud endodormancy, GA_4_ treatment can promote bud burst (Zhuang et al., 2015). Gibberellic acid (GA) also regulates plant growth and development, including seed germination and bud dormancy (Ogawa et al., 2003; Zheng et al., 2018b). It is believed that GA plays a key role in the release of dormancy in buds, but the molecular mechanism of gibberellin regulation is undefined. Gibberellic acid signaling consists of three important parts, including GA, the GA receptor GID1 (GIBBERELLIN INSENSITIVE DWARF1), and DELLA protein (Fukazawa et al., 2015). Gibberellin signal transduction responds to a series of signals through the GA-GID1-DELLA pathway (Hirano et al., 2008). The GID1 receptor belongs to the Hormone Sensitive Lipase (HSL) family and contains motifs for HGG and GDSSG which can bind to GA (Gazara et al., 2018). Without GA binding, the N-terminal extension (N-Ex) of GID1 has a flexible structure that is highly sensitive to protease treatment. The binding of GA to the thC-terminal domain of GID1 induces a conformational switch of its N-Ex to cover the GA-binding pocket (like closing the lid). This binding also creates hydrophobic surfaces for DELLA binding and changes the N-terminal domain, which promotes binding to DELLA, and thus inhibits GA signaling (Sun, 2010). The identification of GID1 in rice was the first elucidation of a protein responsive to GA signaling (Ueguchi-Tanaka et al., 2005). There are three GID1 (*AtGID1a, AtGID1b*, and *AtGID1c*) genes in *Arabidopsis* as GA receptors (Nakajima et al., 2006), and GID1b also interacts with DELLAs under low GA levels. A rice GID1 suppressor mutant reveals that GA is not always required for the interaction between its receptor, GID1, and DELLA proteins. This suggests that GA-dependent or GA-independent pathways induce GA stimulation in growth and development (Yamamoto et al., 2010).

According to the phenotype of the gibberellin insensitive dwarf1mutant, the GID1 family genes are likely partially redundant in *Arabidopsis* (Gallego-Giraldo et al., 2014a). Gibberellin insensitive dwarf1 genes generally control fruit set and fruit growth in *Arabidopsis* (Gallego-Giraldo et al., 2014b), while GID1a primarily regulates growth and GID1b and GID1c play an important role in *Arabidopsis* seed development (Gallego-Giraldo et al., 2014a). Gibberellin insensitive dwarf1ac and GID1b play distinct roles in *Brassicaceae* seed germination (Voegele et al., 2011). The rice GID1 mutant exhibits a dwarf phenotype (Ueguchi-Tanaka et al., 2005). In cucumber (*Cucumis sativus*), *CsGID1a* is essential for fruit locule formation (Liu et al., 2016). There are two GID1 genes in peach that are similar to GID1b and GID1c in *Arabidopsis* (Hollender et al., 2016). The silencing of the GID1c gene in peach results in dwarfing (Hollender et al., 2016; Cantín et al., 2018; Cheng et al., 2019). Moreover, a recent study indicated that the GID1 gene might serve a role in the release of peach vegetative bud dormancy (Hollender et al., 2016).

The peach originated in China and has been cultivated for about 3,000 years (Zheng et al., 2014). Today, peaches (*Prunus persica*) are widely cultivated and recognized as economically important deciduous fruit across the world (Cao et al., 2016). The production of peaches is dependent on the breakage of bud endodormancy, but the mechanism of endodormancy release in peaches is still unclear. Thus, understanding the mechanism of bud endodormancy is of vital importance to control the breaking of buds. In this study, we aimed to characterize the expression patterns of GA-GID1-DELLA genes in peach bud endormancy. Together, this study will better inform how we can use the mechanisms of peach bud endormancy to regulate peach production and management.

## Materials and Methods

### Plant Materials and Dormancy Treatments

Experiments were conducted at the Shandong Agricultural University from 2018 to 2019. The annual branches of the peach buds of 10-year-old trees (*P. persica var. nectarina* cv. Zhongyou 4) were used as test materials. Peach bud samples were collected every 15 days or so, immediately frozen in liquid nitrogen (LN), and then stored at −80°C. On December 25, 2018, different types of hormones [GA_3_, GA_4_, GA_5_, abscisic acid (ABA), and FLU: ABA synthesis inhibitor] were used to treat peach branches for 48 h. After 25 days, we observed the burst rates of the peach leaf buds. Gibberellins, FLU, and ABA were purchased from Thermo Fisher (Shanghai, China). The use of hydroponics to determine the burst rates is the most common method to define the endodormancy process (Li et al., 2011). First, we selected robust annual peach branches every 15 days, inserted them into the water, and placed them in a light incubator. The culture conditions were 25°C, light for 16 h, and dark for 8 h with a 200-μmol·m^−2^·s^−1^ light intensity treatment. Every 2 to 3 days, the base was cut off, and the bud burst rate was measured after 25 days. When the burst rate reached more than 50%, the endodormancy was considered released (Wang et al., 2015).

### RNA Extraction and Quantitative Real-Time PCR

Total RNA was isolated from 0.5 g of bud tissue using an RNAprep Pure Plant Kit (Tian Gen, Beijing, China) according to the instructions of the manufacturer. A NanoPhotometer P360 (Implen, Munich, Germany) was used to assess the quality and quantity of the RNA. First-strand cDNA was generated using a HiScript Q RT SuperMix for qPCR (+gDNA wiper) (Vazyme, Nanjing, China) according to the instructions of the manufacturer. The product was used either immediately in the next reaction or stored at −20°C. Quantitative real-time PCR (qRT-PCR) was performed using SYBR Premix Ex Taq (Takara) on a CFX96 real-time PCR detection system (Bio-Rad). The *PpUBQ* gene was used as the internal control (Supplementary 1). Three biological replicates were used for each analysis. The PCR protocol was as follows: pre-denaturation at 95°C for 2 min, 40 cycles at 95°C for 15 s, 60°C for 30 s, and 72°Cfor 40 s. When the reaction was complete, we proceeded to the dissociation curve reaction. The 2^−ΔΔCT^ method was used to estimate the relative expression level (Livak and Schmittgen, 2001). The Statistical Analysis GraphPad Prism software (GraphPad Software Inc., San Diego, CA, USA) was used to construct charts. The statistical analysis was performed using the IBM SPSS Statistics 19 software (IBM Corporation, New York, USA) to analyze the significance of the differences among data, with a significance level of *p* < 0.05 under Duncan's test.

### Gene Isolation and Bioinformatic Analysis

Based on the previous results, there were two gibberellin insensitive dwarf1 genes identified in the peach genome. Prupe.6G332800 (*Ppa018174*) was referred to as *PpGID1c* in peaches, which shared 95.3% similarity with *AtGID1c* (Hollender et al., 2016). The structure of the *PpGID1* genes was analyzed using TBtools (Chen C. et al., 2018). According to previous research reports, GID1 protein sequences from a total of eight species were obtained, and all protein sequences were downloaded from the TAIR (http://arabidopsis.org/), Phytozome (https://phytozome.jgi.doe.gov/pz/portal.html), and RGAP websites (http://rice.plantbiology.msu.edu/index.shtml). The sequence alignment was performed using the DNAMAN software (Lynnon Biosoft, Foster City, CA, USA). The phylogenetic tree was built with MEGA6 by employing the neighbor-joining method (Tamura et al., 2013).

### Liquid Chromatography-Tandem Mass Spectrometry

Extraction steps: Grind the peach bud sample in LN until it is crushed, accurately weigh all the samples into a test tube, add 10 ml of acetonitrile solution add 2 μl of internal standard mother liquor, extract overnight at 4°C, centrifuge at 12,000 g for 5 min, and take the supernatant. Then, add five times the volume of acetonitrile solution to the precipitate again, extract two times, and combine the supernatants. Afterward, add 35 mg of C18 filler, shake vigorously for 30 s, centrifuge at 10,000 g for 5 min, and take the supernatant. Blow-dry with N and reconstitute with 200 μl of methanol dissolve, pass through a 0.22-μm organic phase filter membrane, and put it in a refrigerator at −20°C to be tested on the machine.

Liquid phase conditions: column: poroshell 120 SB-C18 reversed-phase chromatography (2.1 × 150, 2.7 um); column temperature: 30°C; mobile phase: A:B = (methanol/0.1% formic acid) (water/0.1% formic acid); injection volume: 2 μl; mass spectrometry parameters: ionization mode: electrospray ionization (ESI) positive and negative ion mode monitoring separately; scan type: multiple reaction monitoring (MRM); curtain gas: 15 psi; spray voltage: +4,500 V, −4,000 V; atomizing gas pressure: 65 psi; auxiliary gas pressure: 70 psi; atomization temperature: 400°C. Selected reaction monitoring conditions for deprotonated GA_4_ [(M-H)-]: substance name: GA_4_; parent ion: 331.1 (m/z); product ion: 243.2^*^/213.1(m/z); declustering voltage: −131 (V); collision energy: −24/−39 (V).

### Construction of the *PpGID1c* Overexpression Vector and Plant Transformation

We cloned the full-length Open Reading Frame sequence of *PpGID1c* (Supplementary 2). A homologous recombination technique (Puchta, 2002) was used to connect it to the PRI-GFP (35S: GFP) vector. The expression vector was then transformed into *Agrobacterium tumefaciens* strain GV3101, while selected monoclonal *Agrobacterium* colonies were used to screen for positive clones by PCR. Transgenic *Arabidopsis* seeds were obtained by the floral dip method (Clough and Bent, 2010). The harvested seeds were screened with 50 mg·L^−1^ of kanamycin (Kan). Resistant seedlings grew normally without chlorosis, while we used PCR to screen positive plants. We selected plants with high target gene expression in *Arabidopsis* as candidates in the T1 generation, while T2 generation seeds were segregated for further selection. Seeds that did not appear to segregate in the T3 generation were selected as homozygous strains; after qRT-PCR identification, at least three high expression homozygous strains were obtained, which were named *PpGID1c*-ox1, *PpGID1c*-ox3, and *PpGID1c*-ox5, and the phenotypes were observed and recorded.

### Yeast Two-Hybrid Assay

The CDS (coding sequence) sequences of the *PpGID1c, PpDELLA1*, and *PpDELLA2* genes were ligated to the pGBKT7 and pGADT7 plasmid backbones by homologous recombination technology, respectively, to generate the BD-*PpGID1c*, AD-*PpDELLA1*, and AD-*PpDELLA2* fusion plasmid vectors (restriction enzyme sites: NdeI and EcoRI). Co-transform Yeast Two-Hybrid yeast competent cells with different combinations were spread on SD/-Leu/-Trp medium (synthetic defined minimal medium without leucine and tryptophan), inverted at 30°C for 2–3 days, had positive clones picked then verified by PCR and diluted to an Optical Density equal to about 0.02, and then inoculated to SD/-Ade/-His/-Leu/-Trp (synthetic defined minimal medium without adenine, histidine, leucine and tryptophan) solid culture plates for interaction detection between *PpGID1c* and *PpDELLA*.

## Results

### Isolation and Characterization of *PpGID1*

**Figure 1:**
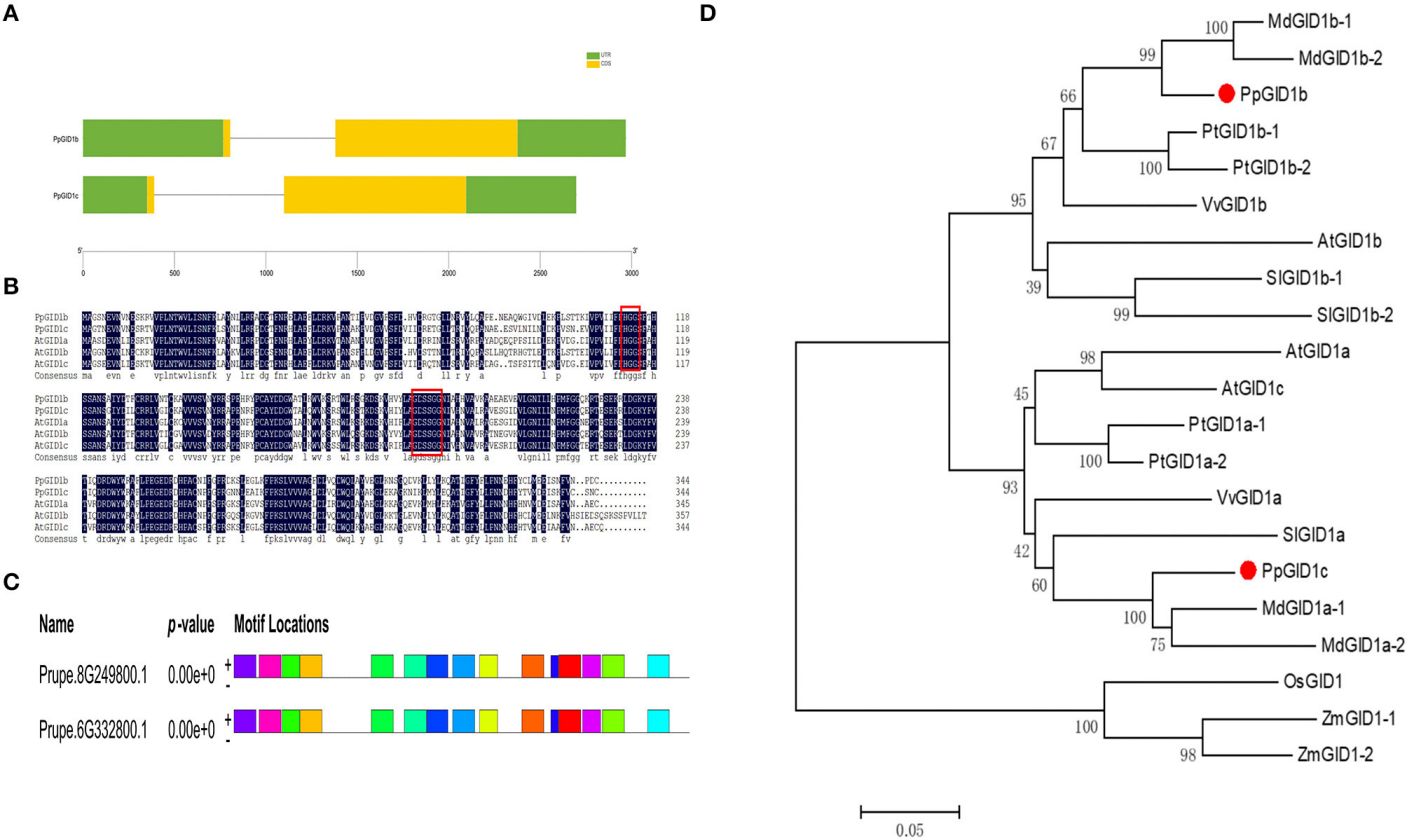
Structure and protein sequence analyses of PpGID1b and PpGID1c. **(A)** *PpGID1b* and *PpGID1c* gene structure. The yellow parts indicate exons, the green parts indicate the CDS sequences, and the lines indicate introns. **(B)** Alignment of the amino acid sequences of PpGID1b and PpGID1c with AtGID1a, AtGID1b, and AtGID1c. The red boxes are marked with HGG and GDSSG domains. **(C)** Analysis of PpGID1 conserved elements. **(D)** Phylogenetic tree of gibberellin insensitive dwarf1 (GID1) proteins from different species. *Prunus persica*: Prupe.8G249800 (PpGID1b), Prupe.6G332800 (PpGID1c). *Populus trichocarpa*: Potri.005G040600 (PtGID1a-1), Potri.013G028700-(PtGID1a-2), Potri.002G213100 (PtGID1b-1), Potri.014G135900 (PtGID1b-2). *Vitis vinifera*: GSVIVG01022014001 (VvGID1a), GSVIVG01011037001 (VvGID1b). *Arabidopsis thaliana*: AT3G05120 (AtGID1a), AT3G63010(AtGID1b), AT5G27320 (AtGID1c). *Solanum lycopersicum*: Solyc01g098390 (SlGID1a), Solyc06g008870 (SlGID1b-1), Solyc09g074270 (SlGID1b-2). *Oryza sativa*: LOC_Os05g33730 (OsGID1). *Zeamays:* GRMZM2G173630_T01 (ZmGID1-1), GRMZM2G016605_T02 (ZmGID1-2). *Malusdomestica*: MDP0000319301(MdGID1a-1), MDP0000445131 (MdGID1a-2), MDP0000319522 (MdGID1b-1), MDP0000929994(MdGID1b-2). *PpGID1b* and *PpGID1c* are marked with red dots.

To further explore the function of the peach gibberellin insensitive dwarf1 gene, a BLAST comparison analysis of the GID1 protein sequence in *Arabidopsis* was performed, which revealed two similar GID1 genes in the peach gene database. Based on the similarity to the GID1 protein sequence in *Arabidopsis*, we named Prupe.6G332800 and Prupe.8G249800 as *PpGID1c* and *PpGID1b*, respectively (Supplementary 3). *PpGID1c* consists of a 2,698-bp genomic region and encodes 344 amino acids. A gene structure analysis found that both the *PpGID1b* and *PpGID1c* genes contain two exons and one intron (Figure 1A). Sequence alignments were carried out with the GID1 genes between peach and *Arabidopsis*. The *PpGID1* protein had high similarity with other *Arabidopsis* GID1 proteins, and it also has conserved HGG and GDSSG domains. This conserved domain is the key site for gibberellin receptors to sense gibberellin (Figure 1B). MEME (http://meme.nbcr.net/meme/cgi-bin/meme.cgi) and Pfam (http://pfam.sanger.ac.uk/) were used to analyze the conservative structure of *PpGID1*, which found that *PpGID1c* and *PpGID1b* had similar motif gene structures, indicating that the GID1 gene is highly conserved in peaches (Figure 1C). To analyze the phylogenetic relationship among the GID1 genes, we collected GID1 genes from eight species according to a study by Yoshida et al. (2018) (Supplementary 4). Phylogenetic analysis also revealed the evolutionary relationship of GID1 homologs in different species and that the peach GID1c is highly similar to the apple GID1 protein, which all belong to the *Rosaceae* species (Figure 1D).

### GA_4_ Levels and the Expression of *PpGID1* During Peach Leaf bud Dormancy

**Figure 2:**
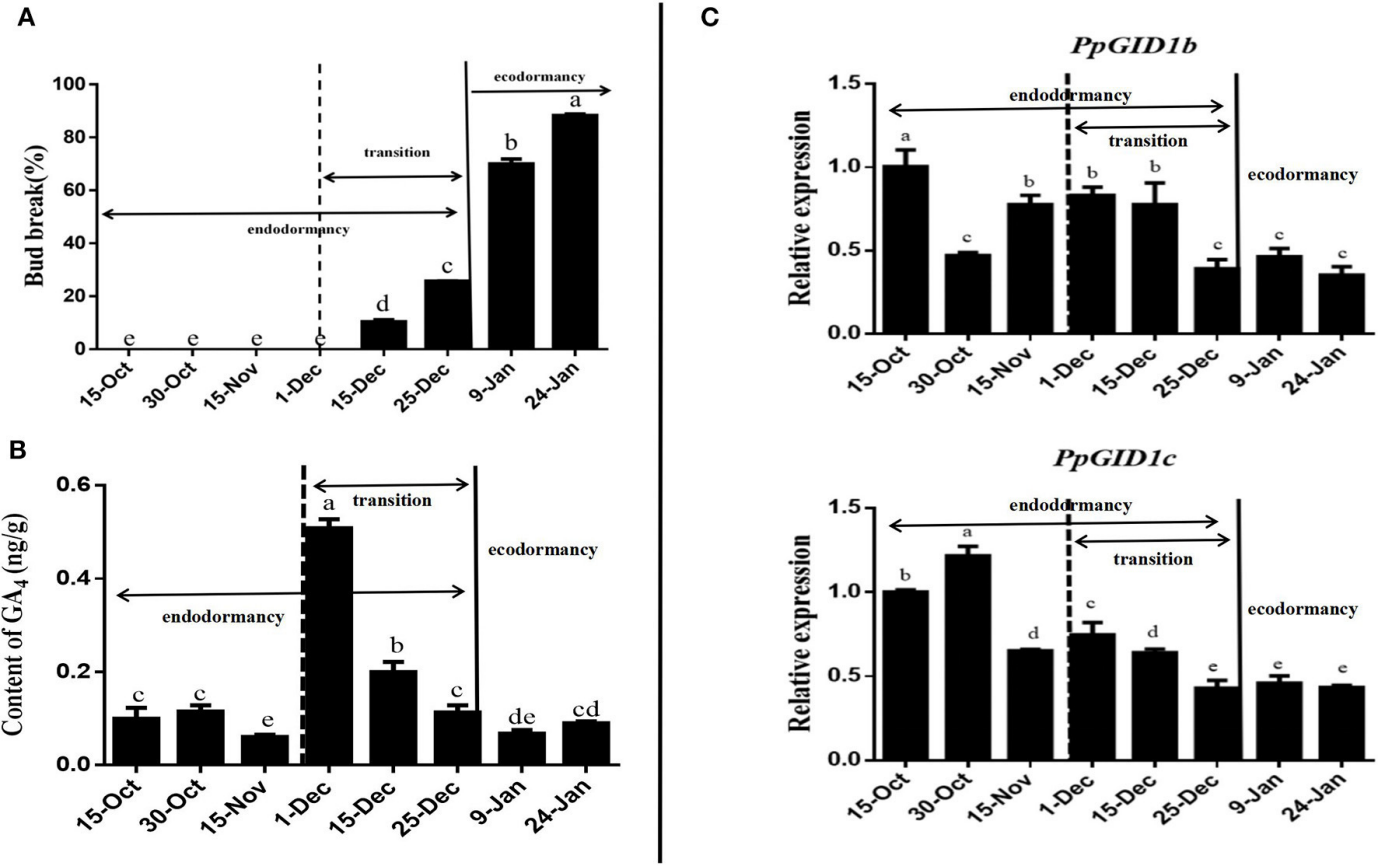
Determination of dormancy progress, GA_4_ levels, and the expression of *PpGID1*. **(A)** Bud-break percentage of the Zhongyou 4 variety from October 15, 2018, to January 24, 2019. Annual shoots were collected from peach trees in the field on the dates indicated and placed in water for 25 d before assessment. Shoots with <50% bud-break were considered dormant, and the values are means of 20 shoots. **(B)** GA_4_ levels in leaf bud determined by liquid chromatography-tandem mass spectrometry (LC-MS/MS). **(C)** Relative expression of *PpGID1b* and *PpGID1c* during peach leaf bud dormancy. Data are means (± *SD*) of the three biological replicates, and expression is relative to that of *PpUBQ*. Different letters indicate significant differences between means as determined by an ANOVA followed by Duncan's multiple range test (*P* < 0.05).

To determine whether the expression of *PpGID1* is related to peach leaf bud endodormancy, we first defined the dormancy status of the peach leaf buds from 2018 to 2019 (Supplementary 5). As shown in Figure 2A, from October 15 to December 1, the peach leaf buds did not burst after 25 days of *in vitro* culture and were in endodormancy. From December 1, the peach leaf buds began to burst. On December 25, the leaf bud burst rate was 25.7%, which was still lower than 50%, indicating that the buds were still in endodormancy. Thus, it was in endodormancy from October 15 to December 1, with the endodormancy transition occurring from December 1 to 25. This transition stage is a critical period for relieving endodormancy. The burst rate of leaf buds exceeded 50% from January 9 to 24 of the following year. The main factor limiting leaf budburst at this stage was the low outer temperature, which was the period of ecodormancy.

Next, we determined GA_4_ levels and the *PpGID1* expression pattern during peach leaf bud dormancy (supplementaries 6–8). As shown in Figure 2B, throughout the dormant period, the GA_4_ content of the peach leaf buds showed the tendency to increase first before decreasing, which reached the highest on December 1st. The GA_4_ content of the endodormancy release period was significantly higher than that in the ecodormancy period, which was similar to observations in poplar (Karlberg et al., 2010; Rinne et al., 2011). In addition, the expression level of *PpGID1b* and *PpGID1c* during ecodormancy was significantly lower than endodormancy. Furthermore, during the period of endodormancy release (December 1st to 25th), the expression of *PpGID1c* was positively correlated with changes in GA_4_ levels (Supplementary 9), suggesting that *PpGID1c* may have a promoting effect on the endodormancy release of peach leaf bud (Figure 2C).

### Heterologous Overexpression of *PpGID1c* in *Arabidopsis*

**Figure 3:**
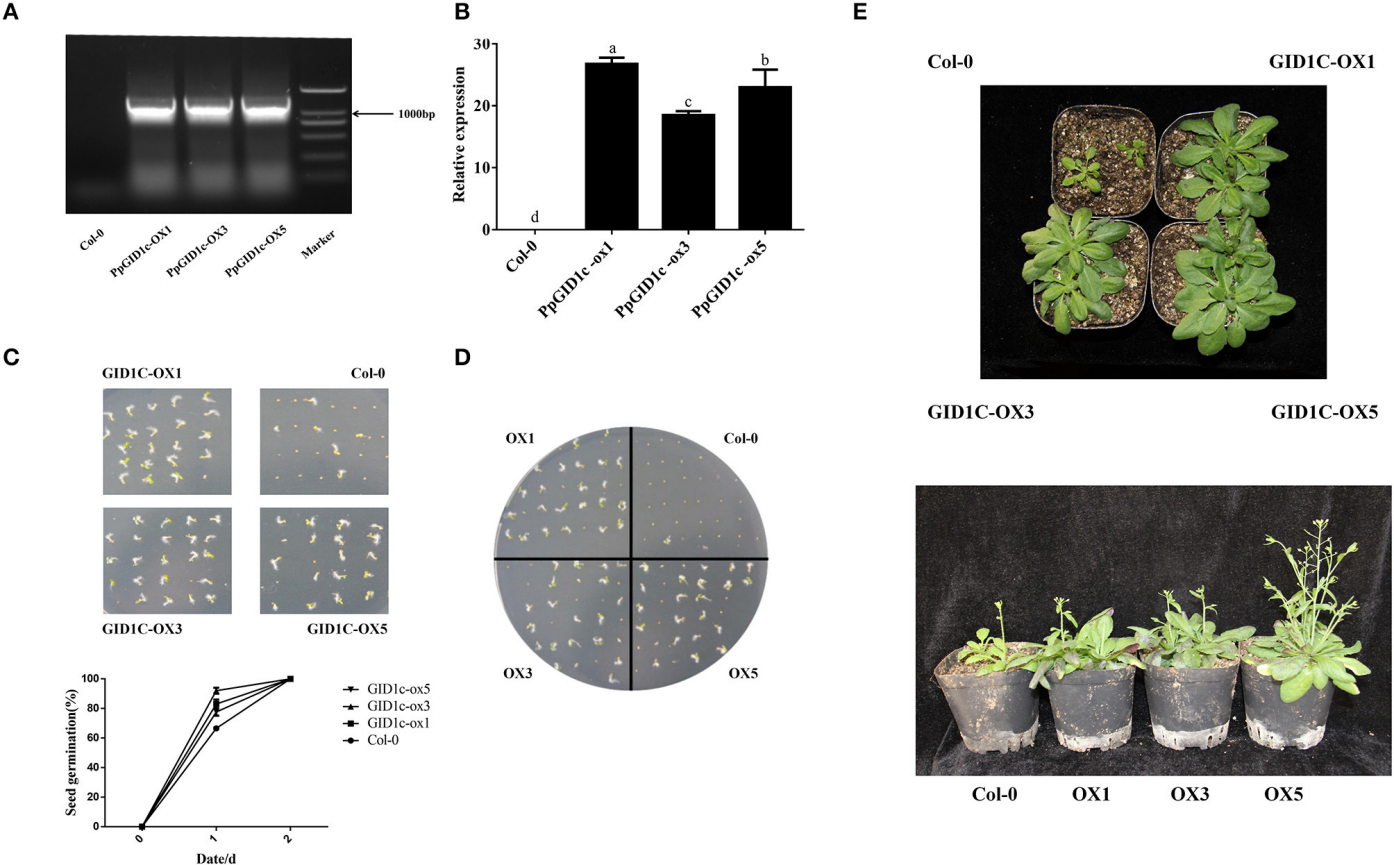
*PpGID1c* transgenic *Arabidopsis* line overexpression identification. **(A)** PCR identification of T3 generation positive transgenic lines. **(B)** Quantitative real-time PCR (qRT-PCR) identification of T3 generation positive transgenic lines. **(C)** Determination of germination rate of Col-0 and *PpGID1c* transgenic *Arabidopsis* in an MS medium. **(D)** Determination of the germination rates of Col-0 and *PpGID1c* transgenic *Arabidopsis* seeds in MS + PAC. **(E)** Phenotyping of rosette leaves and flowering in Col-0 and *PpGID1c* transgenic *Arabidopsis*.

Due to the difficulty in obtaining transgenic peach plants, to gain further insight into the function of *PpGID1c*, the 35S::PpGID1c fusion plasmid was heterologously transformed into *Arabidopsis*. The transgenic *Arabidopsis* lines were identified by PCR and qRT-PCR (Figures 3A,B). To elucidate the roles of *PpGID1c* in endodormancy, the homozygous *Arabidopsis* seeds were plated on an MS medium and the germination rate of *PpGID1c* and Col-0 seeds was observed. Compared with Col-0, *PpGID1c* transgenic seeds showed a higher germination rate on day 1 and all were germinated on day 2 (Figure 3C). When GA biosynthesis inhibitor PAC was added to the MS medium, the Col-0 seeds could not germinate, but *PpGID1c* transgenic *Arabidopsis* seeds could relieve this inhibition, which showed the phenomenon of promoting seed germination (Figure 3D). It can be seen that *PpGID1c* transgenic *Arabidopsis* can receive a stronger GA signal, which is beneficial to the promotion of seed germination.

Next, we observed the effect of *PpGID1c* overexpression on the growth and development of *Arabidopsis*. As shown in Figure 3E, the *PpGID1c-*overexpression *Arabidopsis* rosette leaves became longer and wider, with the growth vigor being obviously stronger than Col-0. As the plant grew, the *PpGID1c-*overexpression *Arabidopsis* height became higher compared with Col-0, while the number of inflorescences and branches increased significantly. It also bloomed earlier (Figure 3E).

In summary, the *PpGID1c* promotes *Arabidopsis* seed germination and growth, suggesting that the *PpGID1c* may have the effect of accelerating the release of endodormancy.

### Effects of Exogenous Hormones on the Release of Endodormancy in Peach Leaf Buds

**Figure 4:**
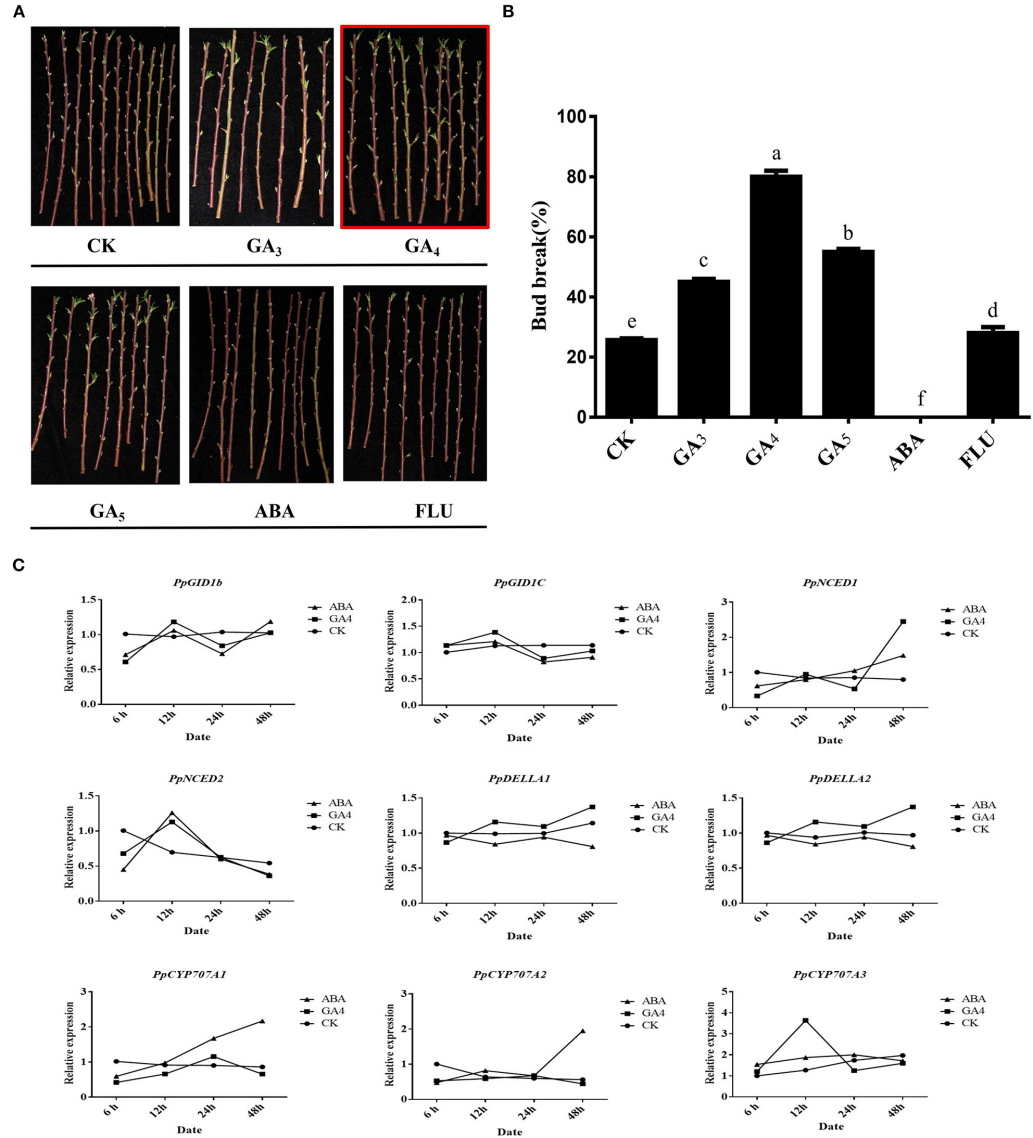
Effects of different hormone treatments on the burst of peach leaf buds. **(A)** Effects of different hormone treatments (GA_3_, GA_4_, GA_5_, ABA, and FLU) on the burst of leaf buds. **(B)** Calculation of peach leaf buds breaks rate. **(C)** Effects of GA_4_ and ABA treatments on GA and ABA signaling pathway gene expression.

Phytohormones play an important role in the dormancy–growth cycle (Horvath et al., 2003; Ruttink et al., 2007). Among them, ABA and GA are the two most important hormones that antagonistically regulate bud endodormancy induction, maintenance, and release (Wang et al., 2015). A high level of endogenous ABA is the primary factor in maintaining bud endodormancy (Zheng et al., 2018a), while GA is responsible for endodormancy release (Zhuang et al., 2013). To analyze the effects of different hormones on peach leaf buds during endodormancy, we used a water treatment as CK, while different types of hormones were used to treat the annual peach branches (Figure 4A). The break rate of the peach leaf buds cultured in water was 25.6%, which was <50%, indicating that it was still in endodormancy. The application of GA could make the break rate reach more than 50%, indicating that GA can promote the release of endodormancy. It also indicated that GA_4_ treatment had the most obvious effect on promoting the bud break, indicating that GA_4_ may be the key gibberellin in promoting the release of peach leaf bud endodormancy (Figure 4B). In addition, ABA could significantly inhibit the bud break of peach leaf buds. Under treatment with fluridone, an ABA biosynthesis inhibitor promoted the endodormancy release in peach leaf buds, suggesting that ABA plays a crucial role in endodormancy maintenance, which is consistent with studies on poplar (Azeez et al., 2021).

The dynamic changes in gibberellin and abscisic acid synthesis and decomposition jointly regulate the process of plant dormancy. As the GA_4_ and ABA treatments had significant effects on peach leaf bud breakage, we further explored the expression of gibberellin signaling- and ABA metabolism pathway-related genes (Supplementary 10). DELLA family proteins play a key role in plant GA signal transmission (Zheng et al., 2015). CYP707A family genes are the coding genes of ABA 8'-hydroxylase, while 9-cis-epoxy carotenoid dioxygenase (NCED) is the key rate-limiting enzyme for ABA biosynthesis in plants; they are the key genes in the process of ABA synthesis and catabolism and have been shown to be involved in peach bud endodormancy (Li et al., 2018). As shown in Figure 4C, the *PpDELLAs* showed opposite expression patterns during the GA_4_ and ABA treatment. GA_4_ treatment promoted the expression of *PpDELLAs* and *PpNCED1*. Abscisic acid treatment downregulated the expression of *PpDELLAs* and upregulated the expression of *PpCYP707A1/2*. It is worth noting that the PpDELLA1 expression trend was consistent with CK after 48 h of GA_4_ treatment. However, not only did the expression of PpDELLA2 not decrease but it increased significantly. These results indicate that GA_4_ mainly affects the expression of *PpDELLA2* and then promotes endodormancy release in peach leaf buds, although this is unreported.

### The Interaction Between *PpGID1c* and *PpDELLA*s

**Figure 5:**
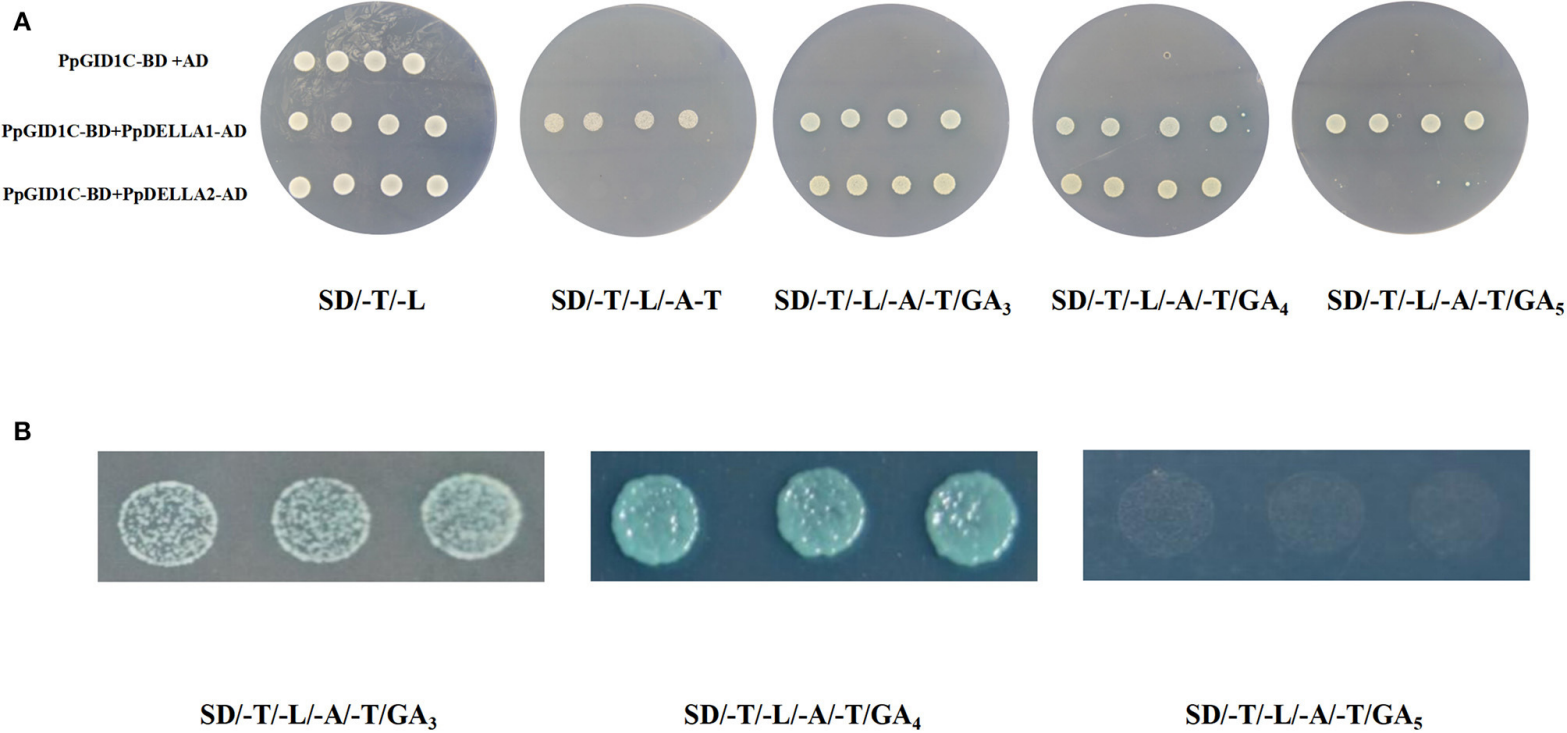
Interaction between PpGID1c and PpDELLA1/2. **(A)** The Y2H-Gold yeast strain was co-transformed with the bait and prey to SD/-Leu/-Trp and SD/-Leu/-Trp/-His/-Ade/GA medium. Yeast cells transformed with AD + BD-PpGID1c were included as negative controls. **(B)** Co-transformed the PpGID1c-BD and PpDELLA2-AD yeast strain in the first 48 h of SD/-Leu/-Trp/-His/-Ade/GA medium.

Two DELLA proteins were identified in the peach genome, which all contain the DELLA and GRAS domains. We named them PpDELLA1 and PpDELLA2. Next, we tested whether the PpGID1c interaction with PpDELLA proteins requires gibberellin. We first tested whether there was an autoactivation of PpGID1c. As shown in Figure 5A, PpGID1c-BD and AD empty vectors can grow normally on SD/-L/-T solid medium but cannot grow on SD/-A/-H/-L/-T solid medium, indicating there was no autoactivation of PpGID1c. Then, we performed an interaction detection with different combinations. Regardless of the presence of GA, PpGID1c-BD and PpDELLA1-AD can grow normally on SD/-A/-H/-L/-T solid medium. In contrast, PpGID1c-BD and PpDELLA2-AD cannot grow on SD/-A/-H/-L/-T solid medium without GA. However, when GA_3_ or GA_4_ is present, PpGID1c can interact with PpDELLA2 and grow normally. It is worth noting that the SD/-A/-H/-L/-T solid medium added with GA_4_, the yeast with the combination of PpGID1c-BD and PpDELLA2-AD, had the best growth rate and morphology in the first 48 h (Figure 5B). These results indicate that the GA_4_-PpGID1c-PpDELLA2 model plays an important role in the endodormancy release period of peach leaf buds.

## Discussion

The seasonal dormancy in deciduous fruit trees is a complex physiological state that is regulated by many plant hormones and genes (Cooke et al., 2012). Gibberellic acid is known to be particularly important in controlling dormancy (Zhuang et al., 2013; Wen et al., 2016). A study by debeaujoni (2000) reported that GA-deficient *Arabidopsis* mutants have increased dormancy and require exogenous GA to germinate, with the removal of the seed coat possibly relieving this need because the seed coat germination barrier and the embryo dormancy caused by ABA need GA to overcome. Gibberellic acid can also improve bud breakage depending on the status of bud dormancy (Zheng et al., 2018a). A previous study in peach found that different GA can promote the breakage of floral bud dormancy (Reinoso et al., 2002). Moreover, GA_3_ and GA_4_ play different roles in *Populus*, as GA_4_ can induced bud breakage while GA_3_ cannot (Rinne et al., 2011). In this study, the different GA that we tested all could promoted leaf bud endodormancy release during the treatments. We also found that, compared with other GA treatments, GA_3_ treatment will cause the severe shedding of peach leaf buds, with the unshed leaf buds becoming <50% (Figure 4). Gibberelic acid_4_ treatment has a significant effect on promoting the burst of leaf buds in the endodormancy period of peaches. In addition, the GA_4_ content of peach leaf buds during the endodormancy release period was significantly higher than in the ecodormancy period. The content of GA_4_ was also positively correlated with the expression of *PpGID1c* (Supplementary 9), which leads us to believe that GA_4_ is the key gibberellin to promote the release of endodormancy.

Previous studies have found that *PpGID1* may be related to peach bud burst (Hollender et al., 2016) and that the GID1 gene has the function of promoting the release of *Arabidopsis* seed dormancy (Amber et al., 2015). The ectopic expression of the *Pinus tabulaeformis* GID1 gene in *Arabidopsis* can promote the germination of seeds (Du et al., 2017). In rice, the overexpression of *OsGID1*-enhanced seed germination and plant growth and development; it also exhibited a GA-overdose phenotype (Chen L. et al., 2018). The overexpression of the GID1c of *P. salicina* can partially compensate for the dwarf phenotype of the Arabidopsis *gid1a-gid1c* double mutant (El-Sharkawy et al., 2014). The Arabidopsis *gid1b-gid1c* double mutant seeds show a phenotype of enhanced dormancy or a failure to germinate (Voegele et al., 2011). In this study, we also found that the *PpGID1c*-overexpression was observed to promote *Arabidopsis* seed germination (Figure 3C), and can relieve the inhibition of PAC compared with Col-0 seeds (Figure 3D). As the plant grows, *PpGID1c*-overexpressing promoted *Arabidopsis* growth and flowering (Figure 3E), suggesting that the GID1c gene might be functionally conserved. Moreover, the expression of *PpGID1c* was upregulated during endodormancy and lowered during ecodormancy (Figure 2C). Thus, we further analyzed the Pearson correlation between the content of GA_4_ and the relative expression of *PpGID1c* during endodormancy, endodormancy release, and ecodormancy (Supplemental 12), which indicated that they all had a consistent trend of change in the corresponding period, showing a significant correlation (*p* < *0.0*5). All results indicated that *PpGID1c* has a pivotal role in the release of endodormancy in peach leaf buds.

A study by Silverstone et al. (1998) reported five DELLA genes (RGA, GAI, RGL1, RGL2, and RGL) in *Arabidopsis*. The DELLA proteins are master components of GA signaling, are repressors of plant growth, and are degraded after binding to GAs (Sun, 2010). Gibberellin stimulates seed germination, stem elongation, and flowering by negatively regulating the DELLA repressors of GA responses (Hauvermale et al., 2012). The overexpression of PmRGL2 in poplar delayed the onset of bud dormancy and resulted in dwarf plants relative to wild-type trees.

The DELLA-dependent feedback regulation of GA biosynthesis has been verified in many GA-sensitive and -insensitive mutants (McGinnis et al., 2003; Dill et al., 2004). The gid1 mutant in rice and *Arabidopsis* showed excessive amounts of DELLA proteins and significantly higher concentrations of endogenous GAs compared with wild-type plants (Ueguchi-Tanaka et al., 2005; Griffiths et al., 2006). For example, GA-insensitive Arabidopsis mutants, for example, and the gain-of-function DELLA-mutants such as gai showed impaired germination (Willige et al., 2007). By contrast, loss-of-function DELLA-mutants such as gai-t6 showed enhanced germination (Kucera et al., 2005). In this study, GA_4_ treatment significantly increased the expression of the GA signaling pathway gene *PpDELLA2*. The Y2H assay showed that the interaction between PpGID1c and PpDELLA1 does not depend on GA signal, but the interaction between PpGID1c and PpDELLA2 requires a GA_4_ signal (Figure 5).

**Figure 6:**
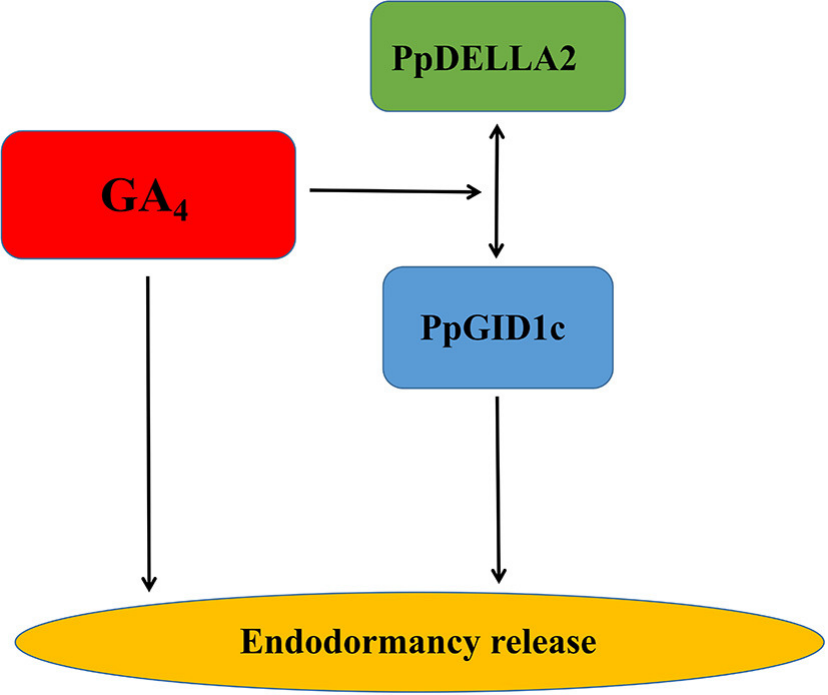
A simple model representing the role of GA_4_-PpGID1c-PpDELLA2 in the regulation of peach leaf bud endodormancy release.

Bud endodormancy is a complex physiological process that is indispensable for the survival, growth, and development of deciduous perennial plants. The timely release of endodormancy is essential for the flowering and fruit production of deciduous fruit trees (Yang et al., 2021). It has been shown that GA_4_ can directly promote the endodormancy release of pear buds (Yang et al., 2019). During endodormancy release, GA_4_ binds to the GID1 receptor, while DELLA binding further stabilizes the GA_4_-GID1-DELLA complex. The complex weakens the inhibitory effect of the DELLA protein on plant growth (Du et al., 2017) where there is an accumulation of the GA_4_-GID1-DELLA complex, which induces the biosynthesis of GA_4_. When GA_4_ levels are high, GA_4_ subsequently mediates the expression of GID1b, GID1c, and DELLA and further regulates the release of endodormancy (Middleton et al., 2012). In short, our research showed that GA_4_ plays a key role in the endodormancy release of peach leaf buds and also provided new insights into the mechanism of action of the GA_4_-GID1c-DELLA2 pathway model in peach leaf bud endodormancy (Figure 6). A better understanding of the mechanism of endodormancy will be of great help in the artificial regulation of endodormancy to cope with climate change and in creating new cultivars with different chilling requirements.

## Data Availability Statement

The original contributions presented in the study are included in the article/Supplementary Material, further inquiries can be directed to the corresponding author.

## Author Contributions

LL, QT, and SL designed the study. SL, QT, and LL performed the experiments and analyzed the data. SL wrote the paper. LL and QT are the co-correspondences. All authors contributed to the article and approved the submitted version.

## Funding

This work was partially supported by (1) the National Natural Science Foundation of China (No. 31872041); (2) National key research and development plan (No. 2018YFD1000104); (3) Shandong Province Agricultural Good Seed Project grant, Nos. 2020LZGC007 and 2020LZGC00702; (4) Funding for major agricultural application technology innovation projects in Shandong Province; (5) Provincial Natural Science Foundation of Shandong (No. ZR2018MC023).

## Conflict of Interest

The authors declare that the research was conducted in the absence of any commercial or financial relationships that could be construed as a potential conflict of interest.

## Publisher's Note

All claims expressed in this article are solely those of the authors and do not necessarily represent those of their affiliated organizations, or those of the publisher, the editors and the reviewers. Any product that may be evaluated in this article, or claim that may be made by its manufacturer, is not guaranteed or endorsed by the publisher.
