# Supplementary material for: Endodormancy Release Can Be Modulated by the GA4-GID1c-DELLA2 Module in Peach Leaf Buds
Source: Front Plant Sci. 2021 Sep 27;12:713514. doi: 10.3389/fpls.2021.713514 (PMC8504481; doi:10.3389/fpls.2021.713514)
Supplement: Supplementary file 2 [file Table_2.docx]

1、PpGID1b

>Prupe.8G249800.1 CDS

ATGGCTGGCAGTAATGAAGTCAACGTCAATGAATCCAAGAGGGTCGTTCCGCTTAATACATGGGTACTCATCTCCAATTTCAAGCTAGCTTACAATCTCC

TGCGCCGGGCTGATGGAACATTCAACCGTGAGTTGGCAGAGTTTCTTGACCGCAAAGTCCCTGCCAATACAATTCCAGTTGATGGGGTTTTCTCATTTGA

TCACGTTGATAGAGGTACCGGACTCCTTAACCGGGTTTACCTACAGGCCCCTGAAAATGAGGCTCAATGGGGGATTGTGGATCTTGAGAAACCCTTGAGC

ACCACCAAGATTGTCCCAGTCATAATTTTCTTCCATGGTGGTAGCTTCACTCATTCCTCAGCCAACAGTGCCATCTATGATACATTCTGTCGCCGTCTTG

TTAATACATGCAAGGCTGTTGTAGTGTCAGTAAATTATCGTCGATCGCCTGAACATCGATATCCTTGTGCATATGATGATGGCTGGGCAACTCTCAAGTG

GGTTAAATCAAGAACATGGCTTCGGAGTGGGAAGGACTCAAAGGTTCATGTTTACCTGGCTGGAGACAGTTCAGGTGGCAACATTGCTCACCATGTTGCA

GTAAAAGCAGCTGAAGCAGAAGTTGAGGTATTGGGAAACATCCTTCTTCACCCCATGTTTGGTGGGCAAAAGAGAACAGAATCAGAAAAGAGATTGGATG

GGAAGTATTTCGTTACAATTCAAGACCGCGATTGGTACTGGCGAGCTTTTCTTCCTGAAGGAGAAGACAGAGACCACCCAGCATGTAATATATTTGGCCC

CAGAGATAAAAGCCTTGAAGGGCTCAAATTCCCCAAAAGTCTTGTTGTTGTGGCTGGTTTTGATCTTGTCCAAGATTGGCAATTGGCGTATGTGGAAGGG

CTGAAGAATTCAGGTCAGGATGTGAAGCTCCTTTATCTAAAGCAGGCCACAATCGGTTTCTACTTCCTGCCAAACAATGAGCATTTCTATTGTCTCATGG

AGGAGATAAGCAACTTCGTCAATCCTGACTGTTAA

1. PpGID1c

>Prupe.6G332800.1 CDS

ATGGCTGGGACCAACGAAGTCAACGTTAATGAATCCAGGACGGTGGTTCCATTGAATACATGGGTCCTCATCTCCAATTTCAAGTTGTCTTACAATCTTC

TTCGTCGACCTGATGGGACTTTTAACCGTCACTTGGCAGAATTCCTTGATCGGAAAGTGCCAGCCAATGCAAAACCAGTTGATGGGGTTGTCTCATTTGA

TGTCATCATTGACCGTGAAACTGGCCTGCTTACTCGAATCTATCAACCAGCCAATGCTGAAGAATCTGTGCTGAATATTCTTAATCTTGACAAACCTGTG

AGCAATGAGGTGGTGCCTGTCATAATTTTCTTCCATGGTGGAAGCTTTGCACACTCCTCTGCTAACAGTGGCATATATGATATTCTGTGCCGCCGACTAG

TTGGTATTTGCAAGGCTGTAGTGGTCTCTGTAAATTACCGCCGGGCACCTGAAAATCGATTTCCTTGTGCCTATGATGATGGATGGACAGCCCTGCAGTG

GGTCAACTCTAGATCGTGGCTTAAAAGTACAAAGGACTCAAAAGTTCATATATATCTTGCTGGTGATAGCTCTGGTGGGAACATTGTACACAATGTTGCT

TTAAGAGCAGTAGAATCTGGAATTGATGTATTGGGAAATATACTGCTCAACCCAATGTTTGGGGGGCAGGAGAGAACTGAATCTGAGAAGCGATTGGACG

GGAAATACTTTGTCACCATCCAAGACCGGGACTGGTATTGGAGAGCTTTTCTCCCTGAAGGGGAAGACAGGGACCACCCGGCATGTAACCCATTTGGTCC

AAGGGGTAATAACCTTGAAGCTATCAAGTTCCCAAAGAGTCTTGTCGTGGTGGCTGGTTTGGATCTTGTTCAGGACTGGCAATTGGCTTATGCTAAAGGG

CTTGAGAAGGCTGGCAAAAACATCAAACTTATGTATCTTGAGCAGGCCACAATTGGTTTCTACTTGCTGCCAAATAATGACCATTTCTACACCGTGATGG

ATGAGATAAGTAAATTTGTGTGTTCCAACTGTTAA
