# Supplementary material for: Endodormancy Release Can Be Modulated by the GA4-GID1c-DELLA2 Module in Peach Leaf Buds
Source: Front Plant Sci. 2021 Sep 27;12:713514. doi: 10.3389/fpls.2021.713514 (PMC8504481; doi:10.3389/fpls.2021.713514)
Supplement: Supplementary file 3 [file Table_3.docx]

>PpGID1b

MAGSNEVNVNESKRVVPLNTWVLISNFKLAYNLLRRADGTFNRELAEFLDRKVPANTIPVDGVFSFDHVDRGTGLLNRVY

LQAPENEAQWGIVDLEKPLSTTKIVPVIIFFHGGSFTHSSANSAIYDTFCRRLVNTCKAVVVSVNYRRSPEHRYPCAYDD

GWATLKWVKSRTWLRSGKDSKVHVYLAGDSSGGNIAHHVAVKAAEAEVEVLGNILLHPMFGGQKRTESEKRLDGKYFVTI

QDRDWYWRAFLPEGEDRDHPACNIFGPRDKSLEGLKFPKSLVVVAGFDLVQDWQLAYVEGLKNSGQDVKLLYLKQATIGF

YFLPNNEHFYCLMEEISNFVNPDC

>PpGID1c

MAGTNEVNVNESRTVVPLNTWVLISNFKLSYNLLRRPDGTFNRHLAEFLDRKVPANAKPVDGVVSFDVIIDRETGLLTRI

YQPANAEESVLNILNLDKPVSNEVVPVIIFFHGGSFAHSSANSGIYDILCRRLVGICKAVVVSVNYRRAPENRFPCAYDD

GWTALQWVNSRSWLKSTKDSKVHIYLAGDSSGGNIVHNVALRAVESGIDVLGNILLNPMFGGQERTESEKRLDGKYFVTI

QDRDWYWRAFLPEGEDRDHPACNPFGPRGNNLEAIKFPKSLVVVAGLDLVQDWQLAYAKGLEKAGKNIKLMYLEQATIGF

YLLPNNDHFYTVMDEISKFVCSNC

>AtGID1a

MAASDEVNLIESRTVVPLNTWVLISNFKVAYNILRRPDGTFNRHLAEYLDRKVTANANPVDGVFSFDVLIDRRINLLSRVYRPAYADQE

QPPSILDLEKPVDGDIVPVILFFHGGSFAHSSANSAIYDTLCRRLVGLCKCVVVSVNYRRAPENPYPCAYDDGWIALNWVNSRSWLKSKKDSKVHIFLAG

DSSGGNIAHNVALRAGESGIDVLGNILLNPMFGGNERTESEKSLDGKYFVTVRDRDWYWKAFLPEGEDREHPACNPFSPRGKSLEGVSFPKSLVVVAGLD

LIRDWQLAYAEGLKKAGQEVKLMHLEKATVGFYLLPNNNHFHNVMDEISAFVNAEC

>AtGID1b

MAGGNEVNLNECKRIVPLNTWVLISNFKLAYKVLRRPDGSFNRDLAEFLDRKVPANSFPLDGVFSFDHVDSTTNLLTRIYQPASLLHQT

RHGTLELTKPLSTTEIVPVLIFFHGGSFTHSSANSAIYDTFCRRLVTICGVVVVSVDYRRSPEHRYPCAYDDGWNALNWVKSRVWLQSGKDSNVYVYLAG

DSSGGNIAHNVAVRATNEGVKVLGNILLHPMFGGQERTQSEKTLDGKYFVTIQDRDWYWRAYLPEGEDRDHPACNPFGPRGQSLKGVNFPKSLVVVAGLD

LVQDWQLAYVDGLKKTGLEVNLLYLKQATIGFYFLPNNDHFHCLMEELNKFVHSIEDSQSKSSPVLLTP

>AtGID1c

MAGSEEVNLIESKTVVPLNTWVLISNFKLAYNLLRRPDGTFNRHLAEFLDRKVPANANPVNGVFSFDVIIDRQTNLLSRVYRPADAGTS

PSITDLQNPVDGEIVPVIVFFHGGSFAHSSANSAIYDTLCRRLVGLCGAVVVSVNYRRAPENRYPCAYDDGWAVLKWVNSSSWLRSKKDSKVRIFLAGDS

SGGNIVHNVAVRAVESRIDVLGNILLNPMFGGTERTESEKRLDGKYFVTVRDRDWYWRAFLPEGEDREHPACSPFGPRSKSLEGLSFPKSLVVVAGLDLI

QDWQLKYAEGLKKAGQEVKLLYLEQATIGFYLLPNNNHFHTVMDEIAAFVNAECQ
